# Global Genetic Population Structure of *Bacillus anthracis*

**DOI:** 10.1371/journal.pone.0000461

**Published:** 2007-05-23

**Authors:** Matthew N. Van Ert, W. Ryan Easterday, Lynn Y. Huynh, Richard T. Okinaka, Martin E. Hugh-Jones, Jacques Ravel, Shaylan R. Zanecki, Talima Pearson, Tatum S. Simonson, Jana M. U'Ren, Sergey M. Kachur, Rebecca R. Leadem-Dougherty, Shane D. Rhoton, Guenevier Zinser, Jason Farlow, Pamala R. Coker, Kimothy L. Smith, Bingxiang Wang, Leo J. Kenefic, Claire M. Fraser-Liggett, David M. Wagner, Paul Keim

**Affiliations:** 1 Department of Biological Sciences, Northern Arizona University, Flagstaff, Arizona, United States of America; 2 Biosciences, Los Alamos National Laboratory, Los Alamos, New Mexico, United States of America; 3 Department of Environmental Studies, Louisiana State University, Baton Rouge, Louisiana, United States of America; 4 The Institute for Genomic Research, Rockville, Maryland, United States of America; 5 Lanzhou Institute of Biological Products, Lanzhou, China; 6 Pathogen Genomics Division, Translational Genomics Research Institute, Phoenix, Arizona, United States of America; Baylor College of Medicine, United States of America

## Abstract

Anthrax, caused by the bacterium *Bacillus anthracis*, is a disease of historical and current importance that is found throughout the world. The basis of its historical transmission is anecdotal and its true global population structure has remained largely cryptic. Seven diverse *B. anthracis* strains were whole-genome sequenced to identify rare single nucleotide polymorphisms (SNPs), followed by phylogenetic reconstruction of these characters onto an evolutionary model. This analysis identified SNPs that define the major clonal lineages within the species. These SNPs, in concert with 15 variable number tandem repeat (VNTR) markers, were used to subtype a collection of 1,033 *B. anthracis* isolates from 42 countries to create an extensive genotype data set. These analyses subdivided the isolates into three previously recognized major lineages (A, B, and C), with further subdivision into 12 clonal sub-lineages or sub-groups and, finally, 221 unique MLVA15 genotypes. This rare genomic variation was used to document the evolutionary progression of *B. anthracis* and to establish global patterns of diversity. Isolates in the A lineage are widely dispersed globally, whereas the B and C lineages occur on more restricted spatial scales. Molecular clock models based upon genome-wide synonymous substitutions indicate there was a massive radiation of the A lineage that occurred in the mid-Holocene (3,064–6,127 ybp). On more recent temporal scales, the global population structure of *B. anthracis* reflects colonial-era importation of specific genotypes from the Old World into the New World, as well as the repeated industrial importation of diverse genotypes into developed countries via spore-contaminated animal products. These findings indicate humans have played an important role in the evolution of anthrax by increasing the proliferation and dispersal of this now global disease. Finally, the value of global genotypic analysis for investigating bioterrorist-mediated outbreaks of anthrax is demonstrated.

## Introduction

Anthrax, caused by the bacterium *Bacillus anthracis*, is a disease with a natural transmission cycle involving wildlife, livestock, and, occasionally, humans. Recently *B. anthracis* received notoriety for its use as an agent of bioterrorism in the 2001 letter attacks in the United States [1], and an unsuccessful aerosol attack in Japan in 1993 [2]. Prior to its use as a bioterrorism agent, *B. anthracis* was developed as a biological weapon by the governments of several countries, including the United States, the United Kingdom, and the former Soviet Union [3]. Despite the emphasis on its role as an agent of bioterrorism or biological warfare, anthrax has been and continues to be an important global disease of wildlife and livestock. Global dispersal of spores via commodities has been prevalent, such that there are currently endemic anthrax foci on all continents except Antarctica (http://www.vetmed.lsu.edu/whocc/). In the environment, *B. anthracis* primarily exists as a dormant, highly stable spore, which is central to the ecology, evolution, and contemporary weaponization of this pathogen. During the spore phase, which may persist for decades, evolution is static or at least greatly reduced in rate, which limits the amount of genetic diversity found among isolates of this species.

In the past the genetic homogeneity of *B. anthracis* severely compromised efforts to reconstruct its evolutionary history. Two molecular approaches, multiple locus variable number tandem repeat analysis (MLVA) and whole genome single nucleotide polymorphism (SNP) discovery and analysis, have greatly enhanced the identification of genetic markers that help to establish the phylogenetic relationships among *B. anthracis* isolates [4], [5]. For example, Keim *et al.* [4] used eight variable number tandem repeat (VNTR) markers to examine a worldwide collection of over 400 *B. anthracis* isolates and described two major clonal lineages (A and B) and 89 unique MLVA8 genotypes. This same VNTR typing scheme also has been used to examine the diversity of *B. anthracis* in France, [6] Poland, [7], Italy [8], and countries in southern [9] and northern Africa [10]. This process has now been expanded to 15 marker-loci, MLVA15 [11].

Although individual SNPs have limited resolving power relative to MLVA, researchers have used phylogenetic approaches to identify SNPs that efficiently partition bacterial strains into genetic groups consistent with their recognized population structure [3], [11], [12]. Recent whole genome sequencing efforts discovered approximately 3,500 SNPs among five strains of *B. anthracis* [5], [13] (J. Ravel, unpublished). Pearson *et al.* [5] mapped nearly 1,000 of these SNPs across 27 diverse isolates and proposed an extremely robust and conserved phylogenetic model for *Bacillus anthracis*. The conserved distribution of SNPs within the *B. anthracis* phylogenetic tree was reflected in the observation that only a single character conflict (homoplasy) was detected from >25,000 data points. These results indicated that that a select number of SNPs representative of specific branches and nodes in the *B. anthracis* SNP-derived tree would be sufficient to accurately determine the current phylogenetic position of any *B. anthracis* isolate. A working hypothesis was formulated [3] where a small number of canonical SNPs (canSNPs) located at key phylogenetic junctions along the *B. anthracis* SNP tree could replace a tedious genome-wide SNP analysis. This strategy is analogous to the TagSNP concept that has been suggested by the International HapMap Consortium for the human genome[14] that “only a minority of sites need to be examined” to fully capture the genotype information in various conserved regions throughout the genome. CanSNPs in *B. anthracis* represent an extreme example of the TagSNP concept where a single SNP can represent the entire genome of an isolate.

In this study, the canSNP hypothesis for *Bacillus anthracis* was tested against a diverse global collection containing >1,000 isolates. An initial set of 12 canSNPs representing different points in the evolutionary history of *Bacillus anthracis* were queried against DNA preparations from this entire collection. These experiments demonstrate that all of the *B. anthracis* isolates can be placed into one of 12 conserved groups or lineages. The slowly evolving canSNP data set was then coupled to the more rapidly evolving MLVA15 marker set to greatly enhance the resolution beyond the original 89 *B. anthracis* genotypes [4]. The analysis of slowly evolving canSNPs allowed the definition of major clonal lineages in *B. anthracis*, whereas the more rapidly evolving MLVA15 markers elucidated younger population-level structure in the species. We also utilized molecular clock models, based upon simple assumptions and exhaustive whole genome synonymous SNP surveys of representative strains, to estimate the age of major events in the evolution of *B. anthracis*. Collectively, our phylogenetic and molecular clock analyses, as well as information on isolate frequencies and global geographic distribution, facilitate the most comprehensive description to date of the global diversity and historical transmission patterns of this pathogen.

## Results

### Canonical SNP analysis

**Figure 1:**
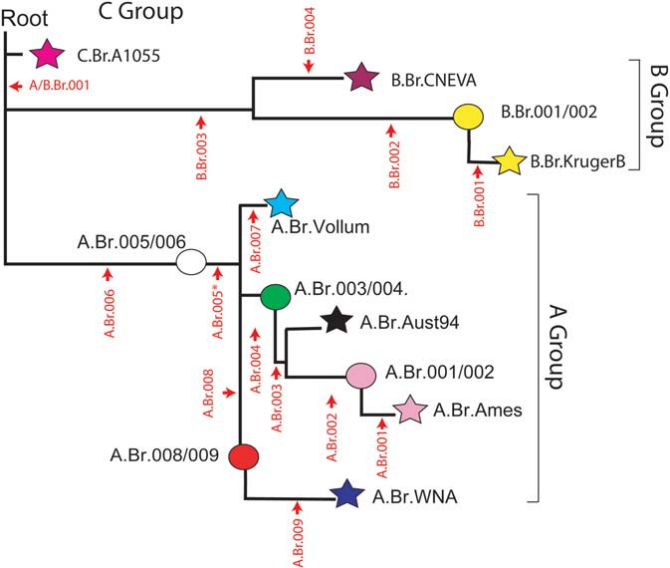
The relationship between canSNPs, sub-lineages and/or sub-groups: The stars in this dendrogram represent specific lineages that are defined by one of the seven sequenced genomes of B. anthracis. The circles represent branch points along the lineages that contain specific subgroups of isolates. These sub-groups are named after the canSNPs that flank these positions. Indicated in red are the positions and names for each of the canSNPs (also see Table 1).

CanSNP analysis subdivided all of the *B. anthracis* isolates into three previously recognized major lineages (A, B and C), with further subdivisions into one of 12 distinct sub-lineages (Figure 1, stars) or sub-groups (circles). Seven completed whole genome sequences (C.USA.A1055, KrugerB, CNEVA.9066, Ames, Australia94, Vollum, Western North America, see Table 1) defined endpoints (stars) that describe 7 distinct sub-lineages within the canonical SNP tree. These seven strains were picked to represent previously recognized diversity within *B. anthracis* [4], [5]. In addition to the 7 lineages the canSNP analysis identified 5 sub-groups labelled as positions along the branches in the canSNP tree. The positions of each of the canSNPs are illustrated in Figure 1 and the canSNP genotype for each of the 7 sub-lineages and the 5 sub-groups is shown in Table 1. It is important to note that all of the 1,033 isolates in this *B. anthracis* collection fell into one of these 12 subdivisions and that the specific sequenced lineage isolates are only representative of a cluster of related isolates within that lineage.

**Table 1:** CANONICAL SNPs.

Lineage/Group	Type Strain.	Sequence	A.Br.001	A.Br.002	A.Br.003	A.Br.004	A.Br.006	A.Br.007	A.Br.008	A.Br.009	B.Br.001	B.Br.002	B.Br.003	B.Br.004	A/B.Br.001
**C.Br.A1055**	C.A1055	C.USA.A1055	T	G	A	T	C	T	T	A	T	G	G	T	**G**
**B.Br.KrugerB**	B1.A0442	KrugerB	T	G	A	T	C	T	T	A	**C**	T	A	T	A
B.Br.001/002	B1.A0102		T	G	A	T	C	T	T	A	**T**	**T**	A	T	A
**B.Br.CNEVA**	B2.A0402	CNEVA.9066	T	G	A	T	C	T	T	A	T	G	A	**C**	A
**A.Br.Ames**	A2.A0462	Ames	**C**	A	G	C	A	T	T	A	T	G	G	T	A
A.Br.001/002	A2.A0034		**T**	**A**	G	C	A	T	T	A	T	G	G	T	A
**A.Br.Aust94**	A1.A0039	Australia94	T	**G**	**G**	C	A	T	T	A	T	G	G	T	A
A.Br.003/004	A2.A0489		T	G	**A**	**C**	A	T	T	A	T	G	G	T	A
**A.Br.Vollum**	A1.A0488	Vollum	T	G	A	T	A	**C**	T	A	T	G	G	T	A
A.Br.005/006	A1.A0158		T	G	A	**T**	**A**	**T**	T	A	T	G	G	T	A
A.Br.008/009	A1.A0293		T	G	A	T	A	T	**G**	**A**	T	G	G	T	A
**A.Br.WNA**	A1.A0193	W. N. America	T	G	A	T	A	T	G	**G**	T	G	G	T	A

CanSNPs and profiles for the lineages/groups: This table lists each of the 12 lineages and groups and indicates the canonical SNPs that help to define each of the sub-lineages and sub-groups (canSNPs that define a particular sub-lineage or sub-group are indicated in yellow). Each lineage is named after the whole genome sequence that is positioned as an end point in a branch created by a comparison of that particular genome sequence to 6 other genomes (stars in Figures 1 and 3). As endpoints all but one of the lineages are defined by a single canSNP (see profiles in yellow for B.Br.Kruger , B.Br.CNEVA, A.Br.Vollum, A.Br.Ames and A.Br.WNA. Although Aust94 is an endpoint the canSNPs that define this lineage were developed before the draft sequence and as a result two canSNPs A.Br.002 and A.Br.003 define the branch point where this isolate is located. Similarly, the groups are positions that define branch points [5], [35] along the different lineages (Circles in Fig. 1 and 3). They carry the group name designations corresponding to the canSNPs that flank these positions and are indicated in blue in this table (e.g. A.Br.001/002). Note that the sub-group need at least two canSNPs (one SNP on either side of the node) to assign a correct sub-group. Sub-group A.Br.005/006 requires three canSNPs to assign an exact genotype because a canSNP for A.Br.005 has not yet been tested. The whole genome sequences for Bacillus anthracis strains A0155, Ames Ancestor, CNEVA-9066, Kruger B, Vollum, Western North America (WNA) and Australia 94 can be found in the NCBI microbial genome website at http://www.ncbi.nlm.nih.gov

### MLVA15 analysis

**Figure 2:**
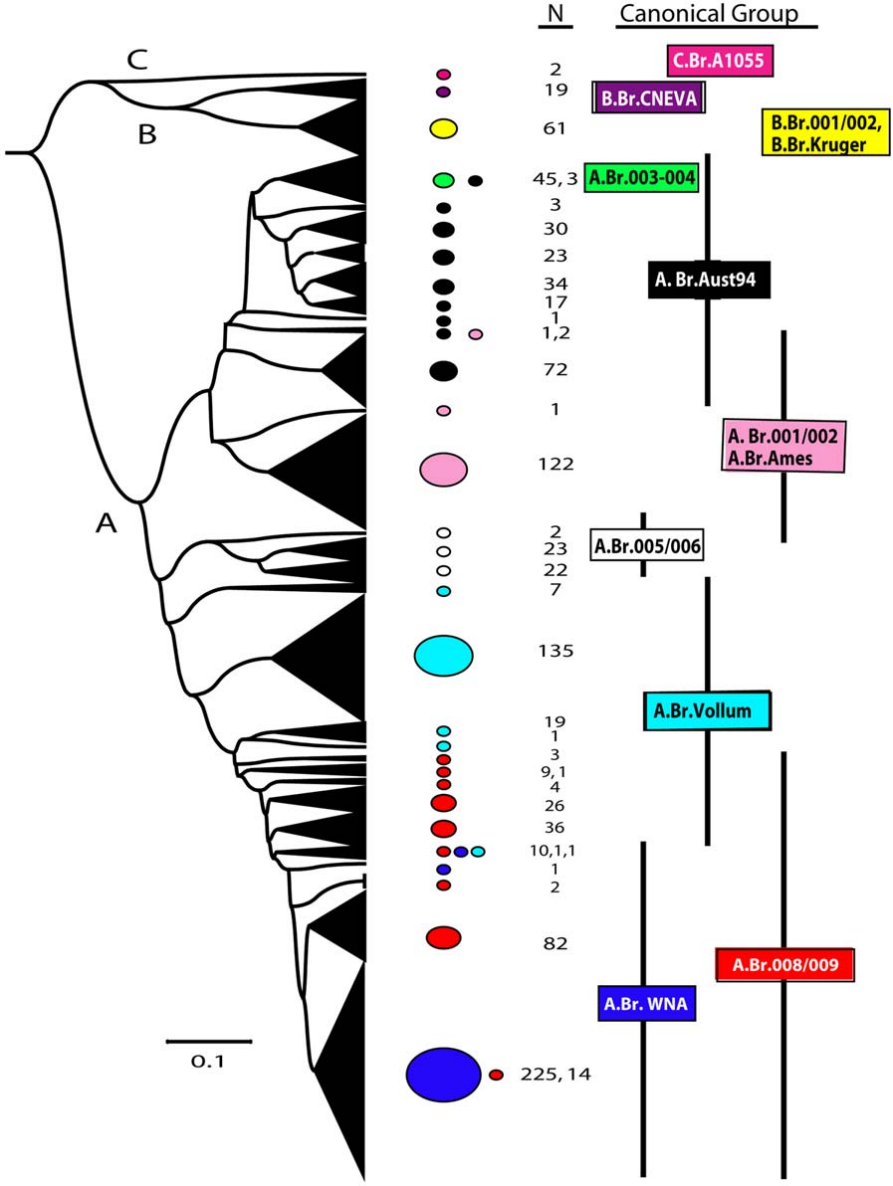
UPGMA dendrogram of VNTR data from worldwide B. anthracis isolates: Fifteen VNTR loci and UPGMA cluster analysis were used to establish genetic relationships among the 1,033 B. anthracis isolates. In this UPGMA dendrogram, which was created using MEGA software [39], groups of genetically similar isolates are collapsed into black triangles that are sized in proportion to the number of isolates in that particular lineage. VNTR loci mutate at faster rates than SNPs and, hence, provide greater resolution for terminal branches. Longer branches, such as the B and C lineages, have length underestimation in this analysis due to mutational saturation. The scale bar indicates genetic distance. Also illustrated on this figure is the distribution of the canonical SNP groups relative to the MLVA phylogeny (right columns). The number of isolates (N) associated with each canSNP group is shown in the second column. The correlation between the phylogenetic clusters identified by the canSNP and MLVA analysis with regards to the world wide geographic distribution of these clusters can be seen in Figure 3.

UPGMA cluster analysis of the MLVA15 data alone clearly identifies the three major genetic lineages (A, B, and C; Figure 2). The longer B and C branch lengths (Figure 1) are underestimated in this analysis (Figure 2) due to mutational saturation of the rapidly evolving MLVA markers. This dataset also increased the number of unique *B. anthracis* MLVA genotypes from 89 (MLVA8, [3]) to 221 owing to both a larger subset of isolates and the expanded resolving power of the MLVA15 marker set (Figure 2, Tables S1 and S2). The MLVA15 tree (Figure 2) illustrates that the majority of isolates are located in shallow branches within the A lineage whereas the B and C lineages have rarer genotypes and fewer isolates. The MLVA15 dataset indicates that 89.6% (198) MLVA genotypes are from the A branch, 10% (22 MLVA genotypes) are from the B branch, and only 0.4% (1 MLVA genotype) are from the C branch.

### Geographical distribution of clonal sub-lineages and sub-groups

**Figure 3:**
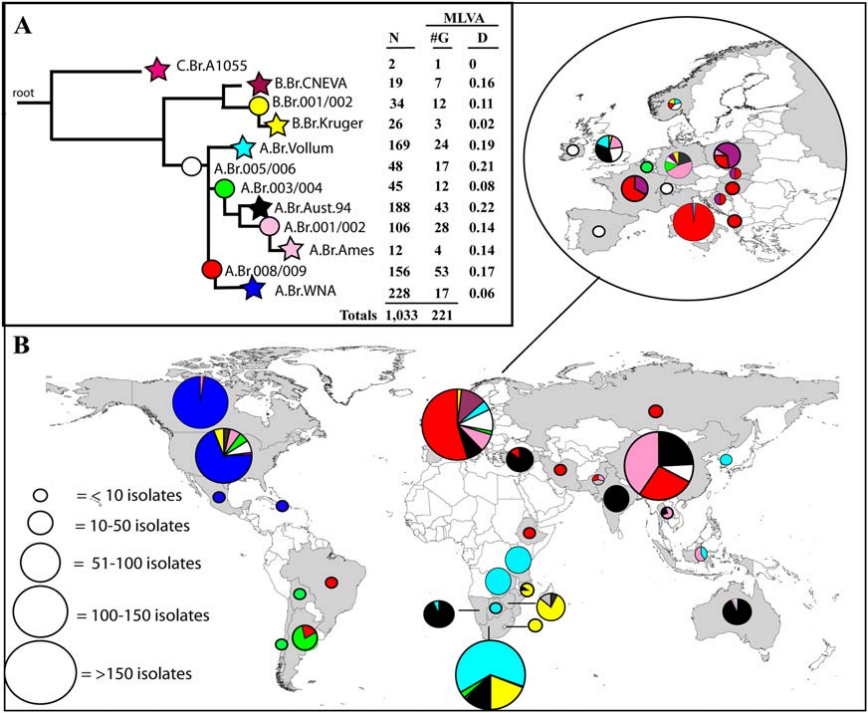
Worldwide distribution of B. anthracis clonal lineages:Phylogenetic and geographic relationships among 1,033 B. anthracis isolates. (A) Population structure based upon analysis of data from 12 canSNP (Protocol S1). The numbers of isolates (N) and associated MLVA genotypes (G) within each sub-lineage are indicated as well as the average Hamming distance (D) as estimated from VNTR data. The major lineages (A, B, C) are labelled, as are the derived sub-lineages (1–12), which are also color-coded. (B) Frequency and geographic distribution of the B. anthracis sub-lineages. The colors represented in the pie charts correspond to the sub-lineage color designations in panel A.

Figures 2 and 3A graphically depict the distribution of the 1,033 isolates into the 12 canSNP sub-lineages and/or sub-groups (Column N in Fig. 2 and 3A) and also indicates the number of unique MLVA15 genotypes that were found in each of the 12 canSNP groupings (Figure 3A, column G; also see Table S1 in the Supplemental Section). The canSNP sub-lineages and sub-groups in Figure 3A also were assigned unique color codes to assist in establishing correlations between these 12 canSNP groupings and the geographic origins of each isolate. These data are presented in Figure 3B as color-coded pie charts for various geographic regions. Each pie chart illustrates the proportion of each canSNP grouping and the total number of isolates that originated from a particular geographic region. North America, Europe, China and parts of Africa are very well represented in these studies, whereas South America and Australia have reasonable representation. Countries from the Middle East and the former Soviet Union are under-represented. These sample biases are important considerations but do not appear to mitigate major genetic and geographic trends in this data set.

There are distinct differences in the global distributions of the major *B. anthracis* clonal lineages (A, B, and C). The A lineage isolates are widely distributed and are found in all countries included in this study. In contrast, the geographic distributions of the B and C lineage isolates are restricted, for example, the B lineage is primarily found in South Africa [B.Br.Kruger B sub-lineage and B.Br.001/002 sub-group [9] and portions of Europe [B.CNEVA-9006 sub-lineage; [4], [6], [7] with geographical differentiation at the sub-group level. Examples of these sub-lineages are rarely found outside of these regions.

Although A branch genotypes appear to be scattered throughout the world, there are distinct subgroup geographic compositions for many regions. The dominant genotypes in Southern Africa, for example, belong to the A.Br.Vollum sub-lineage, whereas in Europe isolates from A.Br.008/009 sub-group are dominant. Although central Asia is poorly represented in our collection, the genetic diversity in Eurasia appears to change along a longitudinal axis. Isolate collections from the west (Europe) are dominated by A.Br.008/009 sub-group isolates, and collections from western and south-central Asia (Turkey, India) and western China are dominated by genotypes belonging to A.Br.Aust94 sub-Lineage, (regional data not shown). Further into central and eastern China the genotypes are dominated by isolates belonging to the A.Br001/002 sub-group and A.Br.Ames sub-lineage (regional data not shown). Distinctive genotype compositions are also observed in the western hemisphere, which is dominated by unique clonal lineages that are not observed in the eastern hemisphere. Within the Americas, North and South America contain different genetic groups of *B. anthracis*: North American genotypes belong mainly to the A.Br.WNA sub-lineage and South American genotypes belong mainly to A.Br.003/004 sub-group.

A striking feature of isolate collections from the Americas is that the dominant clonal groups are rarely observed outside of these regions. These collections also exhibit low genetic diversity even when analyzed using high-resolution MLVA markers (Figure 2). For instance, in South America isolates from the A.Br.003/004 sub-group (mean within-group VNTR distance = 0.08; Fig. 3A) comprise more than 80% of the total isolates from this region yet are rarely observed elsewhere in the world. A similar trend is observed in the more extensive isolate collection from North America, which is dominated (70%) by a single group (sub-lineage A.Br.WNA; mean within-group VNTR distance = 0.06; Fig. 3A) that is not observed outside of North America. In contrast, the dominant sub-lineages in Europe, Asia, and Africa exhibit greater within-group genetic distances [Europe = A.Br.008/009 sub-group, mean within-group genetic distance = 0.17; South Asia (India, Turkey) = A.Br.Aust94 sub-lineage, mean within-group genetic distance = 0.22; East Asia (China) = A.Br.001/002 sub-group, mean within-group genetic distance = 0.14; Southern Africa = A.Br.Vollum sub-lineage, mean within-group genetic distance = 0.19]. In more industrialized regions, such as Western Europe and the United States, we observe dominant clonal lineages but also the co-occurrence of greatly differing genetic types. Important “donor” regions can be identified and differentiated from “recipient” regions based upon their strain diversity and the positions of these strains in phylogenetic models.

### Molecular Clock Estimates

Our models, based upon simple assumptions and whole genome synonymous SNP surveys, allowed us to generate age estimates for the major events in the evolutionary history of *B. anthracis* (Methods). The divergence of the rare C branch isolates from the lineage containing the A and B branches appears to have occurred approximately 12,857 to 25,714 ybp. The more recent divergence of the A and B branch from a common ancestor occurred approximately 8,746 to 17,493 ybp. On a more recent time scale, we estimate that the primary A-radiation in *B. anthracis*, which is clearly evident in Figures 2 and 3A, occurred approximately 3,277 to 6,555 ybp, or in the mid-Holocene (Table 2).

**Table 2:** Molecular clock estimates of separation times among *B. anthracis* sub-lineages.

Compared lineages^a^	Major groupings	Total synonymous sites^b^	Observed sSNPs	sSNP substitution frequency	1 death per year model (ybp±2 STD)^c^	0.5 death per year model (ybp±2 STD)^c^
^d^Vollum /^e^Ames	A vs. A	899,987	153	1.7E-04	3,801±123	7,603±174
^d^Vollum /^f^WNA	A vs. A	899,957	129	1.4E-04	3,205±113	6,411±160
^e^Ames/^f^WNA	A vs. A	902,239	114	1.3E-04	2,825±106	5,651±150
**Average among A branch divergence times = **					**3,277±114**	**6,555±162**
^g^CNEVA/^e^Ames	B vs. A	901,936	322	3.6E-04	7,983±179	15,966±253
^h^KrugerB vs ^e^Ames	B vs. A	902,983	384	4.3E-04	9,509±195	19,019±276
**Average B branch/A branch divergence times = **					**8,746±187**	**17,493±264**
^g^CNEVA/^h^KrugerB	B vs. B	901,935	188	2.1E-04	**4,661±137**	**9,322±193**
^i^C.A1055/ ^g^CNEVA	C vs. B	901,783	484	5.4E-04	**12,002±219**	**24,003±310**
^i^C.A1055/^e^Ames	C vs. A	901,791	553	6.1E-04	**13,713±234**	**27,425±331**

a Sub-lineages according to Fig. 1, bTotal Syn Sites  =  The total sites for synonymous substitutions were determined separately for each pair-wise comparison. c The model for sSNP substitution rate is particularly sensitive to number of death cycles per year. Therefore, two possible scenarios (1 and 0.5 deaths per year) were modelled (see supporting methods on the PNAS website for more details). STD  =  The standard deviation for observed sSNPs, calculated as the square root of the time estimate. Thus, 2 STD represents ∼95% confidence interval based upon fluctuation in this parameter of the model. d Sequence from the Vollum strain, The Institute for Genome Research (TIGR). e Sequence from the ‘Ames Ancestor’ strain, GenBank Reference Sequence NC 007530. f Sequence from the Western North America USA 6153, TIGR.g Sequence from the CNEVA-9066, TIGR. h Sequence from the Kruger B strain, TIGR. I Sequence from A1055, TIGR

## Discussion

*B. anthracis* is thought to have diverged from a *B. cereus* ancestor by the evolutionary acquisition of two virulence plasmids (pX01 and pX02) and several important chromosomal mutations, such as the nonsense mutation in *plcR* [15]–[18]. Subsequent evolution within this pathogen is evidenced by differences in the global distribution and abundance of isolates from the major clonal lineages (A, B, and C). In *B. anthracis*, the more common genotypes and the majority of isolates are located in shallow branches within the A lineage (Figures 2, 3A); whereas the B and C lineages are associated with rarer genotypes and fewer isolates. If isolate abundance is used as a fitness estimator, with rare genotypes considered less fit than common types, genotypes from the C branch and, to a lesser extent, the B branch appear to have very low fitness relative to the A branch genotypes (Figures 2, 3). Indeed, the C branch has significantly slower evolutionary rates than the A branch ([5]; Figure 2), suggestive of fewer infective cycles in nature.

The A branch of *B. anthracis* has experienced a recent and massive radiation (Figures 2 and 3A) that was clearly a very important event in the evolution of anthrax. Evidence for this event includes the great success of the A branch and its clonal derivatives, the involvement of A genotypes in most of the recent anthrax outbreaks around the world, and short phylogenetic branch lengths within this group. This last point is best illustrated in the dendrogram generated from the MLVA data alone (Figure 2), which capitalizes upon the rapid evolution of VNTR loci to depict the recently-derived radiative lineages within the A branch. The domination of A branch genotypes on a global scale is indicative of great reproductive success (hence, fitness) and considerable long-distance dispersal (Figure 3B). In the absence of the A-lineage expansion, anthrax likely would be a highly restricted and rare disease.

There are several possible explanations for the differences in global distribution and abundance observed among the major lineages of *B. anthracis*. One explanation is adaptive genetic differences that affect survival and propagation in either the environment or hosts. A comparison of A vs. B isolates from Kruger National Park, South Africa indicated that A strains were adapted to more diverse environments than B strains, which were restricted to more narrow environmental conditions [9]. This trend is also reflected on a global scale, where the B and C types may be successful locally or regionally but, unlike the A strains, are not a dominant presence worldwide. The limited abundance and geographic distribution of these rarer lineages may arise from fitness costs associated with niche specialization [9], [19].

In addition to possible adaptive differences among lineages, stochastic processes such as human-mediated dispersal may explain the greater success of particular genetic groups. The global genetic population structure of *B. anthracis* suggests human activities have played a role in the proliferation and dispersal of this now global disease and we see evidence for these human impacts on several time scales. For example, models based upon simple assumptions and whole genome synonymous SNP surveys suggest the primary A-radiation in *B. anthracis* occurred approximately 3,277 to 6,555 ybp, or in the mid-Holocene (Table 2). These age estimates coincide with periods of increased human activities in animal domestication and domesticate population expansion [20]–[24]. Although the importance of the development of human civilization and animal domestication in the natural history of anthrax has been recognized [20], [21], our study presents genetic evidence that it dramatically influenced the global population structure of *B. anthracis*.

As an important disease of livestock, it seems logical that major evolutionary events in anthrax, such as the A radiation, coincide with human developments in agriculture, animal domestication, and Old World trade routes. Animal husbandry and farming practices, which forced animals into confined areas, are likely to have increased *B. anthracis* infection and evolutionary rates, which would rapidly increase genotypic diversification. Similarly, the population expansion of large mammal domesticates from the centers of domestication in Eurasia and North Africa would function to disperse *B. anthracis* genotypes. Molecular clock models suggest that African and Eurasian cattle populations expanded 9,000 ybp and 5,000 ybp, respectively [24]; a time period that roughly corresponds to the A lineage radiation (3,277 to 6,555 ybp) and the divergence of the two major B branches from a common ancestor (4,661–9,322 ybp).

Independent domestication and domesticate expansion events may provide an explanation for the different assortments of A and B lineages on these two continents. For example, the two major B lineages are spatially separated, one is found in southern Africa (B.Br.001/002 sub-group and B.Br.KrugerB sub-lineage) and the other (B.Br.CNEVA-9066 sub-lineage) is found in portions of Europe, suggesting that after diverging from a common ancestor, these two groups experienced independent evolutionary histories. The divergence of the B.Br.CNEVA and B.Br.Kruger sub-lineages are similar in molecular clock estimates to the A radiation and, again, could represent human influences on this pathogen. Taken together, human-mediated events in the mid-Holocene provide plausible explanations for both the dramatic events in *B. anthracis* evolution observed during this time period and the diversity among and within clonal lineages on the African and Eurasian landmasses.

The dispersal of *B. anthracis* to the western hemisphere was probably via intercontinental transport of animal products during European colonization [25], [26]. Evidence for this includes isolate collections from the western hemisphere that are dominated by clonal groups that are rarely observed outside of these regions and exhibit low genetic diversity when analyzed using high-resolution markers. These patterns are consistent with single, relatively recent introductions followed by widespread dispersal, ecological establishment, and local differentiation. The close derived genetic relationship between the North American sub-lineage A.Br.WNA and the dominant European sub-group A.Br.008/009 is consistent with an introduction to North America from Europe, possibly via French or Spanish colonization [25], [26].

More recent human activities in commerce and industrialization also appear to have impacted the global population structure of *B. anthracis*. For instance, in addition to a single dominant genetic type, North America also contains a cosmopolitan assortment of rarer *B. anthracis* genotypes that are likely a consequence of international industrial trade (*e.g.,* wool, skins, bone meal, shaving brushes). A similar phenomenon is observed in other industrialized regions, such as Western Europe, where we observe the co-occurrence of greatly differing genetic types. The dispersal of these genotypes to industrialized regions has been tied to the trade of spore-infected items [25], [27]. For instance, in the United Kingdom, the presence of minor genetic types that are dominant in portions of southern and eastern Asia (sub-lineages A.Br.Aust94, A.Br.001/002, A.Br.Ames; Figure 3) is consistent with reports tracing anthrax infections to imported animal products from these regions during the 19^th^ and early 20^th^ century [28]–[31]. Certainly, the highly-stable *B. anthracis* spore plays an important role in the importation of diverse genotypes into industrialized countries via transport and trade of contaminated commodities across large distances.

Trade also seems the likely source of *B. anthracis* in Australia. It has been hypothesized that anthrax was first introduced to Australia in 1847 via contaminated bone meal-based fertilizer shipped from India. Following this initial introduction at Sydney, the disease is thought to have spread along stock routes to the interior of the country [32]. Our genetic data provide some support for this hypothesis. All ten of the isolates we examined from India were assigned to sub-lineage A.Br.Aust94, which also appears as the dominant sub-lineage in Australia. It must be noted that the preponderance of isolates from A.Br.Aust94 lineage in Australia stems in part from a collection that is dominated by isolates from a single anthrax outbreak. Our genetic data, in fact, indicates separate introductions into Australia of isolates that belong to the A.Br.005/006 and A.Br.001/002 sub-groups; sub-groups that are more commonly found in Southern Africa and Eastern Asia, respectively.

*B. anthracis* has been developed as a biological weapon by several nations and terrorists groups and this has greatly increased the value of genotyping analysis for applications that attempt to differentiate between natural and bioterrorist-mediated outbreaks of anthrax. This is illustrated in the identification of the Ames strain as the source for the weaponized material from the 2001 anthrax letter attacks in the USA [1], [3], [13]. We found that the Ames strain genotype, which was originally obtained from a dead cow in Texas in 1981, is unique in this isolate collection and, hence, apparently rare in nature. North America is well represented in this study with 273 isolates spanning 44 MLVA genotypes (A.Br.WNA plus isolates from other sub-lineages, Figure 3B). However, the Ames genotype was present only once (although genetically similar isolates to the Ames strain were also identified in Texas, USA). The rarity of the Ames genotype in nature, coupled with its widespread use as a laboratory strain, makes it unlikely that the source material utilized in the 2001 bioterrorist attack was acquired directly from nature. These findings further highlight the importance of large genetic-geographic databases for distinguishing between intentional and environment-acquired infections caused by organisms that are both potential biological weapons and widespread in the environment [8], [33], [34].

In summary, our analyses of both canSNP and MLVA data provide a description of the global diversity and historical transmission patterns of *B. anthracis*. Our data suggest that although *B. anthracis* is a naturally occurring pathogen, human activities have dramatically influenced its current distribution and occurrence. We observe the effects of human activities at three levels: 1) the massive radiation of the A-branch in the mid-Holocene, 2) the more recent colonial-era importation of specific *B. anthracis* genotypes from the Old World into the New World, which lead to their ecological establishment, and 3) the repeated industrial importation of rare diverse genotypes into developed countries through animal products (e.g. wool, hides, and bone meal). The genetic population structure of *B. anthracis* is indicative of these long distance transmission events and illustrates its ability to become ecologically established in new locations. Fortunately, natural outbreaks of anthrax can be managed effectively through vaccination and public health efforts. However, due to actual and potential nefarious use of the pathogen, anthrax will likely remain of great social and scientific importance.

## Materials and Methods

### Nomenclature

The tree in Figure 1 is based upon an analysis of >1,000 SNPs discovered amongst seven complete or draft genomes of *B. anthracis*, which yielded a branched phylogeny containing seven lineages corresponding to the sequenced “discovery” genomes [5]. In a strictly clonal species, like *B. anthracis*, these genomes will be situated at the end of each branch. These terminal lineages are depicted as stars Figure 1 and each of these lineages is named after the sequenced isolate (e.g. Ames, KrugerB, Vollum, etc.). The canSNPs are named after one of the three main clades (e.g. A, B, or C) followed by a three digit number (A.Br.001, A.Br.002, A.Br.003). Where possible, we have tried to be systematic in naming the canSNPs. For example, the first canSNP in the A branch was proximal to the Ames genome sequence (or the lineage terminus) and is named A.Br.001 (red labels in Figure 1). The second canSNP position defines a position between canSNP A.Br.001 and the circled position called A.Br.001/002. Such a systematic naming scheme for canSNPs may be compromised by future studies that define additional lineages and branches (*i.e.* the order of the canSNPs from the terminus will be inconsistent with their names). Hence, this should only be considered an arbitrary numbering system, but it will function effectively as new phylgenetic discoveries are made. The circles in the dendrogram represent branches or branch points defined by flanking characters (canSNPs). The branch points and the ends of lineages (the circles and stars in Fig 1) encompass all 1,033 *B. anthracis* isolates (ranging from 2 isolates in the C lineage (C.Br.A1055) to 228 isolates in the Western North American lineage (A.Br.WNA). Branch points also have been defined and named by their flanking canSNPs (*e.g.* B.Br.001/002). The near total absence of homoplasy (character conflicts in the tree), coupled with character discovery bias, has caused “branch collapse” in this clonally propagating pathogen [5], [35]. A collapsed branch is still defined by its flanking canSNP characters.

### *B. anthracis* isolates

We examined a global collection of 1,033 *B. anthracis* isolates. Table S3 contains information on the numbers and distribution of strains used in this study. These isolates were obtained from known anthrax cases, environmental sources, or other materials associated with the disease. Our isolate collection is biased toward anthrax outbreaks that occurred in the last several decades and towards countries actively engaged in the international exchange of scientific material. It is important to note that all of the isolates analyzed in this study were shown to possess the *plcR* inactivating mutation as detected by the PCR assay described in Easterday *et al.* [16]. This nonsense mutation is considered essential for maintenance of virulence plasmids and represents a definitive character of *B. anthracis* [16], [36].

### DNA isolation

A 1.0 µl inoculating loop was used to transfer *B. anthracis* colony material into 200 µl of Brain-Heart Infusion broth (Hardy Diagnostics, Santa Maria, CA) within the wells of a sterile, untreated polycarbonate 96-well culture plate (Costar Corning Inc., Acton, MA). The plate was then covered with an adhesive plastic film, placed in a secondary containment device, and incubated overnight at 37°C without shaking. Following incubation, 10.0 µl of broth was transferred to a Microseal™ Polypropylene Microplate (MJ Research, Waltham, MA). The samples were then flash-frozen in 96-well cold block (−80°C) for 15 s and then immediately thawed in a 96-well heat block (96°C) for 15 s. This freeze-thaw cycle was repeated two additional times. The cell lysates were then transferred into a 96-well GV 0.2 µM Durapore Multiscreen Plate (Millipore, Billarica, MA) containing 100 µl of TE (10 mM Tris-HCl [pH 8.0], 1.0 mM EDTA) per well. Cellular debris and spores were removed from the 96-well filter plate by vacuum filtration using a MultiScreen Separations System Manifold (Millipore, Bedford, MA). The filtrate was collected into a 96-well plate and used to support PCR for downstream SNP and MLVA genotyping. The sterility of each sample was confirmed by plating 1.0 µl of each filtrate onto a TSA II 5% Sheep Blood prepared media plate (Becton Dickinson and Company, Cockeysville, MD) and incubating at 37°C for 48 hr.

### Genetic Markers

Two types of genetic markers were used to analyze the *B. anthracis* collection: canonical single nucleotide polymorphisms (canSNPs) and variable number tandem repeats (VNTRs). We used data from the Pearson *et al.* [5] and unpublished genomic sequence data (Ravel *et al.*, unpublished data) to identify canSNPs that can be used for identifying a particular phylogenetic point in the evolutionary history of *B. anthracis*. In total, 2 *B. anthracis* specific SNPs and 12 canSNPs to analyze DNA from the 1,033 *B. anthracis* isolates. CanSNP alleles were determined using TaqMan™ -Minor Groove Binding (MGB) allelic discrimination assays. TaqMan™ MGB probes and primers for the canSNPs were designed using ABI Primer Express software and guidelines, with the exception that allele-specific probe lengths were manually adjusted to match melting temperatures [37]. The genomic location for each of the canSNPs can be found in Table S4 while the probe and primer sequences for each are listed in Table S5. Each 10.0 µl reaction contained 1× ABI Universal Master Mix, 250 nM of each probe, and 600 nM each of forward and reverse primers and 1.0 µl of approximately 350 pg/µl template DNA. For all assays, thermal cycling parameters were 50° C for 2 min., 95° C for 10 min., followed by 40–50 cycles of 95° C for 15 sec and 60° C for 1 min. Endpoint fluorescent data were measured on the ABI 7900.

DNA from the isolates was also analyzed using 15 VNTR loci; eight of these VNTRs are described by Keim *et al.* [4] MLVA8 and the additional 7 markers are described by Zinser [38]. These markers were compiled together into a multiple-locus VNTR analysis (MLVA15) subtyping system (see Protocol S1, Table S6 for details on the markers and methods).

### Phylogenetic analyses

Two basic approaches were used to analyze genetic relationships among the 1,033 *B. anthracis* isolates. First, canSNP and VNTR data were used in a hierarchical approach to analyze phylogenetic relationships: data from the slowly evolving canSNPs loci were used to categorize the isolates into clonal lineages and followed by the use of data from the 15 rapidly-evolving VNTR loci to measure diversity and determine the number of genotypes within each of these clonal categories. This system allowed us to effectively analyze both older phylogenetic relationships and younger population-level structure [3]. Second, we used UPGMA cluster analyses of the MLVA15 data alone to illustrate the global population genetic structure in an unbiased manner. All phylogenetic analyses were conducted using MEGA3 software [39].

### Geographic distribution of clonal lineages

To examine genetic-geographic patterns in *B. anthracis*, we mapped the worldwide distribution of the clonal lineages that were identified by the analysis of the canSNP data.

### Age Estimates

To estimate the age of several events in the evolutionary history of *B. anthracis*, we performed whole genome synonymous SNP comparisons of strains that represent major clonal lineages. We utilized the following equation to estimate the time since pairs of strains last shared a common ancestor:
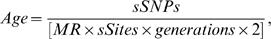
where *sSNPs* is the total number of synonymous SNPs between two strains as determined by whole-genome comparisons, *MR* is the per site synonymous mutation rate in *B. anthracis* (5.2×10^−10^ mutations/generation; [40], *sSites* is the number of synonymous sites in common between the two strains, and *generations* is the estimated number of generations undergone by a given lineage in each year (estimated as 43 per transmission cycle). The number of generations per year is based upon an ungulate transmission model and the number of infection/death cycles per year (see detailed descriptions below). The age estimates are particularly sensitive to the number of infection/death cycles per year. As such, we calculated the estimates using both 1 (43 generations/year) and 0.5 (21.5 generations/year) infection/death cycles per year (Table 2).

### Details of the Age Estimates

The use of sSNPs for the substitution rate restricts these estimates to nearly neutral evolutionary characters. While all SNPs are relatively infrequent among *B. anthracis* isolates, the use of whole genome analysis has identified many sSNPs (Table 1) and resulted in highly robust estimates of relationships among isolates [5]. sSNP occurrence between two strains is modeled well by the Poisson probability distribution. The relative large number of observations makes the error in this estimate small. When the expected number is high, the Poisson become fairly symmetrical with a standard deviation equal to the square root of the expected number. Thus, two standard deviations from the maxima are very close to the 95% confidence interval.

The mutations rates for single nucleotide changes have been reported in *B. anthracis* based upon selection for antibiotic resistance (Rif) and are very similar to the rates observed for other well-studied bacteria (2). In this case, Vogler et al. [40] estimated the rate using the Luria-Delbruck fluctuation test and then partitioned the phenotypic mutation rate (1.55E-09 mutants per generation) to different nucleotide positions in the *rpoB* gene by sequencing this gene in the mutants. Hence, we have a per site mutation rate (5.2E-10 mutations per generation) instead of merely a phenotypic rate.

While Drake [41], [42] has argued for a universal substitution rate for microbial genomes, the extremely episodic nature of anthrax transmission makes this hard to justify among the clonal lineage of *B. anthracis.* Indeed, this is clearly the most sensitive aspect of the substitution rate model with certain parameters highly influential in the final estimates.

### Ungulate transmission model

The number of *Bacillus anthracis* generations (*G*) in a single infected ungulate was determined using the following equation:
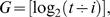
where *t* = terminal number of *B. anthracis* organisms in a 100 kg ungulate (100 kg × *d*), *i* = initial number of *B. anthracis* organisms in the ungulate as obtained from an environmental source (10 organisms), and *d* = terminal density of *B. anthracis* organisms per unit body weight in a mammal (10^8.8^ organisms per kg) [43]. Based on these parameters, it was estimated that *G* = 43.1, which was rounded to 43. The model is not particularly sensitive to this particular parameter. Changing the size of the animal and, hence, the final *B. anthracis* population size is mitigated by the log_2_ transformation. The number of generations is altered only by 3.3 for every 10-fold increase in population size. This has a minimal affect upon the final number of generations.

### Infection/death cycles per year

Estimating the number of infection/death cycles per year is difficult for anthrax. While hundreds or even thousands of individual animals might die in a single outbreak, it is unlikely that these multiple victims are sequential infection/death cycles. Rather, these clusters are likely to be from a single source, or due to environmental induction of the outbreak. For this reason, we believe that the average annual number of death/infection cycles will be one or less, even in the most endemic regions. *B. anthracis* spores are known to survive long periods of time; though very long-term spore survival is unlike to be important in the overall evolutionary rates as the viability does drop with time. In this study, we are primarily interested in the most highly fit branch of *B. anthracis* (A). Its worldwide distribution and fitness argues for a higher rate of transmission, probably close to one infection/death cycle per year. Because this is one of the most sensitive parameters in the model, we have modeled the molecular clock estimates using both 1 and 0.5 deaths per year. These values translate into 43 or 21.5 generations per year when combined with the population size estimates from a typical host (see above).

## Supporting Information

Table S1 The 221 MLVA Genotypes and Associated Can SNPs. The 221 genotypes (1–221, Column A) are organized according to their Keim Genetics Lab ID Designation (“A” number - Column B), prior designations when available (“K” numbers - Column C), their original MLVA8 GenoTyping designation (“GT” numbers: 1–89 - Column D) from Keim et al., (2000) and the alternative strain designations and original source codes for each isolate (Column E). This is followed by the isolate's canSNP lineage/group (Column F, also see Fig 1), two B. anthracis specific SNPs (Columns G and H), the13 canSNP scores (Columns I–U) and the 15 marker MLVA profile for that isolate (Columns W–AK). The first two SNPs (Column G and H) are Bacillus anthracis specific SNPs originally identified in the plcR and gyrA loci and are not part of the canSNP profile. There are 221 unique MLVA genotypes listed in this table.
(0.15 MB XLS)

Table S2 The MLVA Sizing Code. The VNTR alleles for each MLVA marker in Supplemental Table S1 are letter coded according to size to allow these data sets to be utilized by various tree drawing programs. Apparent MLVA fragment sizes vary from instrument to instrument and even with various size standards. Allele codes provide a common designation in the face of this variation. Table S2 provides a code that describes the fragment sizes for these alleles based on analysis on an ABI 3100 Genetic Analyzer (see Protocol S3), a custom made LIZ®-labeled internal size standard (5), and subsequent analysis using Genotyper. The numeral 1 appears as a code when a fragment failed to amplify; eg., an isolate lacking the pXO1 plasmid would not be able to amplify the pXO1.1AAT VNTR marker.
(0.02 MB XLS)

Table S3 Geographical Composition of B. anthracis isolates used in this study
(0.06 MB DOC)

Table S4 CanSNPs Description and Chromosomal Location
(0.03 MB DOC)

Table S5 Canonical SNP Primers/Probes used in molecular typing of B. anthracis
(0.03 MB DOC)

Table S6 15 VNTR loci in the B. anthracis 15 VNTR MLVA system.
(0.04 MB DOC)
