# Supplementary material for: Global Genetic Population Structure of Bacillus anthracis
Source: PLoS One. 2007 May 23;2(5):e461. doi: 10.1371/journal.pone.0000461 (PMC1866244; doi:10.1371/journal.pone.0000461)
Supplement: Table S3 — Geographical Composition of B. anthracis isolates used in this study (0.06 MB DOC) [file pone.0000461.s003.doc]

# Table S3. Geographical Composition of *B. anthracis* isolates used in this study

| **Continent** | **Country** | **No. of isolates** | **Years^1^** | **canSNP groups** |
| --- | --- | --- | --- | --- |
| Africa | Botswana | 2 | 1998 | 5 |
|  | Ethiopia | 1 | ? | 11 |
|  | Lesotho | 1 | 1995 | 3 |
|  | Mozambique | 6 | 1942-1965 | 3,4,8 |
|  | Namibia | 30 | 1983-1993 | 5,8 |
|  | South Africa | 168 | 1936-1998 | 3,4,5,6,7,8 |
|  | Tanzania | 17 | 1999-2000 | 5 |
|  | Zambia | 27 | 1987-1995 | 5 |
|  | Zimbabwae | 14 | 1980-1982 | 3,6,8 |
| Asia | China | 191 | 1947-1993 | 6,8,9,10,11 |
|  | Hong Kong | 1 | 2003 | 9 |
|  | India | 10 | ? | 8 |
|  | Indonesia | 5 | ? | 5,9 |
|  | Iran | 1 | ? | 11 |
|  | Pakistan | 7 | 1961-1978 | 6,9,11 |
|  | Russia | 1 |  |  |
|  | South Korea | 4 | 1994 | 5 |
|  | Thailand | 4 | 1965 | 8.9 |
|  | Turkey | 43 | 1982-1995 | 8,11 |
| Australia | Australia | 30 | 1994-1997 | 5,8,9 |
| Europe | Albania | 6 | ? | 11 |
|  | Belgium | 1 | 2000 | 7 |
|  | Croatia | 2 | 1996 | 2,11 |
|  | France | 21 | 1992-1997 | 2,11 |
|  | Germany | 15 | 1971-1998 | 2,4,6,7,8,9 |
|  | Hungary | 6 | 1998 | 11 |
|  | Ireland | 1 | ? | 6 |
|  | Italy | 57 | 1945-2002 | 2,5,11 |
|  | Norway | 5 | 1976-1993 | 3,5,6,11 |
|  | Poland | 11 | 1962? | 2,9,11 |
|  | Slovakia | 4 | ? | 2,11 |
|  | Spain | 4 |  |  |
|  | Switzerland | 2 | 1978 | 6 |
|  | UK | 26 | 1964-1995 | 3,5,6,8,9 |
|  |  |  |  |  |
| N. America | Canada | 131 | 1962-2002 | 9,11,12 |
|  | Haiti | 1 | ? | 12 |
|  | Mexico | 2 | ? | 12 |
|  | United States | 139 | 1925-2001 | 1,3,6,7,8,9,10,12 |
| S. America | Argentinia | 30 | 1977-2001 | 7,11 |
|  | Bolivia | 1 | 1999 | 7 |
|  | Brazil | 1 | ? | 11 |
|  | Chile | 4 | ? | 7 |
|  |  |  |  |  |
| Total |  | 1033 |  |  |

# ^1^ In some cases isolate sets from a given country had incomplete date information, therefore year intervals were derived from the subset of isolates for which dates of isolation were known.
