# Supplementary material for: Global Genetic Population Structure of Bacillus anthracis
Source: PLoS One. 2007 May 23;2(5):e461. doi: 10.1371/journal.pone.0000461 (PMC1866244; doi:10.1371/journal.pone.0000461)
Supplement: Table S4 — CanSNPs Description and Chromosomal Location (0.03 MB DOC) [file pone.0000461.s004.doc]

**Table S4: CanSNPs Description and Chromosomal Location**

| Name of SNP | Reference location NC_003997.3 Ames | Type^a^ | Base Change | Gene |
| --- | --- | --- | --- | --- |
| A.Br.001 | 182106 | N | C_T | conserved domain protein |
| A.Br.002 | 947760 | S | G_A com | conserved hypothetical protein |
| A.Br.003 | 1493157 | I | G↔A com |  |
| A.Br.004 | 3600659 | N | C↔T | sugar ABC transporter, permease protein, authentic point mutation |
| A.Br.006 | 162509 | N | A_C | gluconate kinase, authentic point mutation |
| A.Br.007 | 266439 | N | T_C com | sensor histidine kinase |
| A.Br.008 | 3947248 | S | T↔G com | 5-nucleotidase family protein |
| A.Br.009 | 2589823 | S | A_G | glycine betaine/L-proline ABC transporter, ATP-binding protein |
| B.Br.001 | 1455279 | I | T_C com |  |
| B.Br.002 | 1056740 | I | G_T com |  |
| B.Br.003 | 1494269 | S | G↔A | thermostable carboxypeptidase 1 |
| B.Br.004 | 69952 | N | C↔T | cell division protein FtsH |
| A/B.Br.001 | 3697886 | N | A_G | membrane protein, putative |

^a^ I= intergenic, S= synonymous, N= non-synonymous, com = complementary strand in the Ames genome in the real time assays
