# Supplementary material for: Global Genetic Population Structure of Bacillus anthracis
Source: PLoS One. 2007 May 23;2(5):e461. doi: 10.1371/journal.pone.0000461 (PMC1866244; doi:10.1371/journal.pone.0000461)
Supplement: Table S5 — Canonical SNP Primers/Probes used in molecular typing of B. anthracis (0.03 MB DOC) [file pone.0000461.s005.doc]

***Table S5: Canonical SNP Primers/Probes used in molecular typing of*** B. anthracis

| CanSNP.Branch | **Primer Sequences 5’🡪3’** | **Probe Sequences 5’🡪3’** |
| --- | --- | --- |
| A.Br.001 | CAAGCGGAACCAAATTTAATCTTT  TTCACCGTACGTCATTGTATAATACG | FAM- ACCGAAA***C***TTGAAGTC - MGB  VIC- AAACCGAAA***T***TTGAAGTC - MGB |
| A.Br.002 | AACGATACCTAAAATCGATAAAG  GGCAGAAGGAGCAAGTAATGTT | FAM- CGCCCA***G***CCTAA-MGB  VIC - CGCCCA***A***CCTAAA -MGB |
| A.Br.003 | GCTACTGTCATTGTATAAAAACCTCCTTT  CGCTTGCCAAGCTTTTTTTC | FAM - TACCTCAA***G***CTTAATTC- MGB  VIC - CTACCTCAA***A***CTTAATTC- MGB |
| A.Br.004 | CCGATACCAGTAAACGACGACAT  CTGGAATTGGTGGAGCTATGGA | FAM- TTGGAATG***C***CCCTAAT- MGB  VIC-CTTTGGAATG***T***CCCTAAT- MGB |
| A.Br.006 | CCGGAAATTGCTATTAGAACGAA  TCCCAATCTAGCGTTTTTAAGTTCA | VIC-CATCGCCT***C***GTGCA-MGB  FAM-CCATCGCCT***A***GTGC-MGB |
| A.Br.007 | TTGGTAACGAGACGATAAACTGAATAA  GCCTTGGATTGGCGATTG | 6FAM-CATCCTTA***C***ATTCAGCT  VIC- CCATCCTTA***T***ATTCAGCTC |
| A.Br.008 | TTCGCAACTACGCTATACGTTTTAGAT  CAAACGGTGAAAAAGTTACAAATATACG | FAM-ATAATTCTTC***G***CCGCTTG-MGB  VIC- ATTCTTC***T***CCGCTTGTT-MGB |
| A.Br.009 | GGCAATCGGCCACTGTTT  GGGTTTCTACTGTGTATGTTGTTAATAAAAAG | VIC- CGGCTTT***A***CTTGCATC-MGB  FAM-CGGCTTT***G***CTTGC-MGB |
| B.Br.001 | TGCATGCTTCTTCTTACAGAGTAGTTAAT  CGGTCATAAAAGAAATCGGTACAA | FAM-CGATACCTTCTTATC***C***TC-MGB  VIC -CGATACCTTCTTATC***T***TC-MGB |
| B.Br.002 | TGTTGCACCTTCTGTGTTCGTT  GTAGTGGCTTCACCGAATGGA | FAM- CGTTACT***G***CTGTTCC-MGB  VIC-AACGTTACT***T***CTGTTCCT-MGB |
| B.Br.003 | CATTTATTCGCATAGAAGCAGATGA  TGTGCCATCAAATAACTCTTTCTCAA | FAM -ACATATCC***A***CTTCACG- MGB  VIC -CATATCC***G***CTTCACG-MGB |
| B.Br.004 | GAAGTTAAGTATCAACCAGCAGAAGAAA  CCGCCGCCTTGAGCTT | VIC-CTTTACTTCTA***C***CATCCC-MGB  FAM-CTTTACTTCTA***T***CATCCC-MGB |
| A/B.Br.001 | GAAGGTCTCCAATTTGGATTTAAAAT  CGTGTGAACCTTTCGGTAAATAGTC | 6FAM-TTTTATTTAG***A***AGATAGCGGC  VIC-TTTATTTAG***G***AGATAGCGGC |
