# Supplementary material for: Global Genetic Population Structure of Bacillus anthracis
Source: PLoS One. 2007 May 23;2(5):e461. doi: 10.1371/journal.pone.0000461 (PMC1866244; doi:10.1371/journal.pone.0000461)
Supplement: Table S6 — 15 VNTR loci in the B. anthracis 15 VNTR MLVA system. (0.04 MB DOC) [file pone.0000461.s006.doc]

**Table S6. 15 VNTR loci in the *B. anthracis* 15 VNTR MLVA system*.***

| Locus | Location  (GenBank accession # : position) | Repeat motif | Multiplex Reaction | Forward Primer Sequence (5’-3’)  Reverse Primer Sequence (5’-3’) | Primer  Conc. (μM) |
| --- | --- | --- | --- | --- | --- |
| BaVNTR12 | C (NC003995 : 2485205-2485318) | TA | 7-1 | NED-CGTACGAAGTAGAAGTCATTAA  GCATATAATTGCACCTCATCTAG | 0.20  0.20 |
| BaVNTR19 | C (NC003995 : 955120-955243) | TAT | 7-1 | 6-FAM-gtgatgaaatcggacaagttaggag  Gaaatattttattaaacatgctttccatcc | 0.15  0.15 |
| BaVNTR35 | C (NC003995 : 2771693-2771806) | GATTGT | 7-1 | PET-AAATAATATGTTCCTTTTGCTG  GTCCTGAAATAAATGCTGAAT | 0.20  0.20 |
| BaVNTR16 | P (NC003981 : 35721-35992) | ACATTTAC | 7-2 | 6-FAM-CTCTTGAAAATATAAAACGCA  GAATAATAAGGGTTCTCATGGTAT | 0.20  0.20 |
| BaVNTR23 | C (NC003995 : 3974824-3975020) | ATGAAAAAGAAA | 7-2 | PET-TTTAGAAACGTTATCACGCTTA  GTAATACGTATGGTTCATTCCC | 0.30  0.30 |
| BaVNTR17 | P (NC003981 : 85403-85787) | ATGTGTAA | 7-3 | NED-TAGGTAAACAAATTTTCGTAATC  GATCGTACAACAGCAATTATCAT | 0.10  0.10 |
| BaVNTR32 | C (NC003995 : 1378983-1379559) | Complex | 7-3 | VIC-AACTGGATCCAGGAGATTATA  GAAACAAGAGCAAACCCAAT | 0.15  0.15 |
| vrrA^1^ | C (NC003995 : 4549439-4549752) | CAATATCAACAA | 8-1 | VIC-CACAACTACCACCGATGGCACA  GCGCGTTTCGTTTGATTCATAC | 0.25  0.25 |
| vrrB1^1^ | C (NC003995 : 4971393-4971621) | 9bp degenerate | 8-1 | VIC-ATAGGTGGTTTTCCGCAAGTTATTC  GATGAGTTTGATAAAGAATAGCCTGTG | 0.20  0.20 |
| CG3^1^ | C (NC003995 : 2730643-2730800) | TAATA | 8-1 | NED-TGTCGTTTTACTTCTCTCTCCAATAC  AGTCATTGTTCTGTATAAAGGGCAT | 0.40  0.40 |
| pXO1-aat^1^ | P (NC003980 : 149273-149398) | AAT | 8-2 | VIC-CAATTTATTAACGATCAGATTAAGTTCA  TCTAGAATTAGTTGCTTCATAATGGC | 0.25  0.25 |
| pXO2-at^1^ | P (NC003981 : 74965-75105) | AT | 8-2 | PET-TCATCCTCTTTTAAGTCTTGGGT  GTGTGATGAACTCCGACGACA | 0.60  0.60 |
| vrrB2^1^ | C (NC003995 : 4971267-4971419) | Complex | 8-2 | 6-FAM-CACAGGCTATTCTTTATCAAACTCATC  CCCAAGGTGAAGATTGTTGTTGA | 0.25  0.25 |
| vrrC1^1^ | C (NC003995 : 4930818-4931397) | Complex | 8-3 | GAAGCAAGAAAGTGATGTAGTGGAC  6-FAM-CATTTCCTCAAGTGCTACAGGTTC | 0.20  0.20 |
| vrrC2^1^ | C (NC003995 : 4930322-4930853) | Complex | 8-4 | PET-CCAGAAGAAGTGGAACCTGTAGCAC  GTCTTTCCATTAATCGCGCTCTATC | 0.20  0.20 |

^1^Originally described in Keim et al, 2000; C, chromosome; P, plasmid. References.
